# Distinct expression profiles of TGF-β1 signaling mediators in pathogenic SIVmac and non-pathogenic SIVagm infections

**DOI:** 10.1186/1742-4690-3-37

**Published:** 2006-06-26

**Authors:** Mickaël J-Y Ploquin, Jean-François Desoutter, Patricia R Santos, Ivona Pandrea, Ousmane M Diop, Anne Hosmalin, Cécile Butor, Françoise Barre-Sinoussi, Michaela C Müller-Trutwin

**Affiliations:** 1 Unité de Régulation des Infections Rétrovirales, Institut Pasteur, Paris, France; 2 Institut Cochin, Département d'Immunologie, INSERM U567, CNRS UMR8104, Université Paris-Descartes, Faculté de Médecine, Paris, France; 3 Tulane National Primate Research Center, Covington Louisiana 70433, Tulane University Health Science Cente, New Orleans Louisiana 70112, USA; 4 Institut Pasteur, Dakar, Senegal; 5 Université Paris 7 – Denis Diderot, France; 6 Unité de Régulation des Infections Rétrovirales, Institut Pasteur, 25 rue du Docteur ROUX, F75724 PARIS Cedex 15, France

## Abstract

**Background:**

The generalized T-cell activation characterizing HIV-1 and SIVmac infections in humans and macaques (MACs) is not found in the non-pathogenic SIVagm infection in African green monkeys (AGMs). We have previously shown that TGF-β1, *Foxp3 *and IL-10 are induced very early after SIVagm infection. In SIVmac-infected MACs, plasma TGF-β1 induction persists during primary infection [1]. We raised the hypothesis that MACs are unable to respond to TGF-β1 and thus cannot resorb virus-driven inflammation. We therefore compared the very early expression dynamics of pro- and anti-inflammatory markers as well as of factors involved in the TGF-β1 signaling pathway in SIV-infected AGMs and MACs.

**Methods:**

Levels of transcripts encoding for pro- and anti-inflammatory markers (*tnf-α, ifn-γ, il-10, t-bet, gata-3*) as well as for TGF-β1 signaling mediators (*smad3, smad4, smad7*) were followed by real time PCR in a prospective study enrolling 6 AGMs and 6 MACs.

**Results:**

During primary SIVmac infection, up-regulations of *tnf-α, ifn-γ *and *t-bet *responses (days 1–16 p.i.) were stronger whereas *il-10 *response was delayed (4^th ^week p.i.) compared to SIVagm infection. Up-regulation of *smad7 *(days 3–8 p.i.), a cellular mediator inhibiting the TGF-β1 signaling cascade, characterized SIV-infected MACs. In AGMs, we found increases of *gata-3 *but not t-bet, a longer lasting up-regulation of *smad4 *(days 1–21 p.i), a mediator enhancing TGF-β1 signaling, and no *smad7 *up-regulations.

**Conclusion:**

Our data suggest that the inability to resorb virus-driven inflammation and activation during the pathogenic HIV-1/SIVmac infections is associated with an unresponsiveness to TGF-β1.

## Background

Progression to AIDS during HIV-1 infection is linked directly to generalized T cell activation, but only indirectly to viral load (VL) [2,3]. Moreover, increased T cell activation levels from the initial stage of infection have a predictive value for AIDS progression even before seroconversion [4,5]. The precise mechanisms leading to the aberrant chronic T-cell activation in HIV-1 infection remain unclear. The study of acute SIV infections in non-human primate models contributes to the understanding of the early virus/host interactions. SIVmac infection in macaques (MACs) best reflects HIV infection in humans. In contrast, SIV infections in natural hosts of SIV, such as African Green monkeys (AGMs), are generally non-pathogenic. During SIVagm infection in AGMs, plasma VLs are similar to those recorded for pathogenic HIV-1/SIVmac infections [6] and SIVagm replicates in lymphoid tissues, including the gut [6,7]. Despite high VLs, natural carriers of SIV do not show increased lymphocyte activation profiles during chronic infection [8]. Our recent data indicate that AGMs are capable of controling T cell activation rapidly after SIVagm infection. This control was associated with the immediate induction of an anti-inflammatory environment [1], including an immediate burst of plasma TGF-β1 [1]. Surprisingly, plasma TGF-β1 was detectable for longer periods of time in SIVmac-infected MACs [1]. Elevated levels of plasma TGF-β1 were also reported in HIV^+ ^patients with chronic, progressive infection [9,10].

TGF-β1 is known to mediate negative regulation of inflammation. We raise the hypothesis that the early burst of TGF-β1 down-modulates inflammation in AGMs, whereas the long lasting plasma TGF-β1 levels reflect the inability of MACs and humans to resorb virus-driven inflammation and activation [1], perhaps because HIV/SIVmac infections would render cells unresponsive to TGF-β1. Therefore we searched for differences between SIV-infected AGMs and MACs at the levels of molecules which mediate the ability to respond to TGF-β1. We found significant differences in the expression levels of activating and inhibitory mediators of the TGF-β1 signaling pathway between pathogenic and non-pathogenic SIV infections.

## Methods

Six Chinese rhesus macaques (*M. mulatta*) and 6 AGMs (*C. sabaeus *from Senegal) were infected intravenously with SIVmac251 and SIVagm.sab92018, respectively [1]. The Central Committee for Animals at Institut Pasteur, Paris, France and the Committee for Ethics and Animal Experimentation at the International School of Science and Veterinary Medicine in Dakar, Senegal, reviewed and approved the use and animals care. This study was conducted on the same animals for which we previously assessed plasma IL-10 and TGF-β1 (active and latent) responses [1]. To get a robust baseline, peripheral blood mononuclear cells (PBMC) were harvested 7 times in each animal before infection with the same sampling schedule as used after infection between days 1 to 13 p.i. PBMC isolation, total RNA extraction from PBMC and reverse transcription were previously described [1]. Quantification of *t-bet, gata-3, smad3, smad4 *and *smad7 *transcripts was performed by using Taqman gene expression assays developed by Applied Biosystems. The references of those assays are Hs00203436_m1, Hs00231122_m1, Hs00706299_s1, Hs00232068_m1 and Hs00178696_m1, respectively. Primers and probes were previously described for *tnf-α*, *ifn-γ *and *il-10 *[1]. The expression of each gene was normalized against the expression of *18S rRNA *used as an endogenous control [1,11]. For each marker, the value at each time point after infection was compared to the individual baseline before infection (Statview, Wilcoxon signed-rank test) [1].

## Results

**Figure 1:**
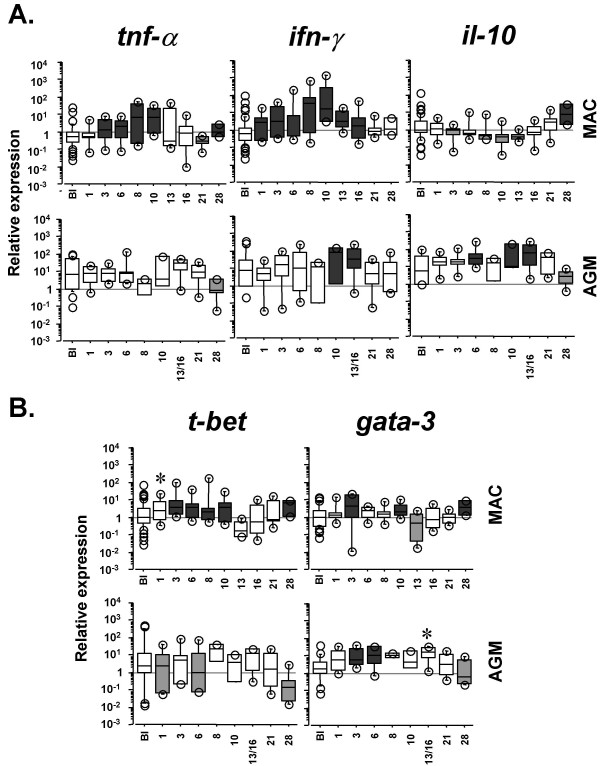
**Dynamics of pro- and anti-inflammatory markers in PBMC during pathogenic and non-pathogenic SIV infections**. **A**. *Tnf-α*, *ifn-γ *and *il-10 *expressions. **B**. *T-bet *and *gata-3 *expressions. Upper and lower panels represent data from 6 SIVmac-infected rhesus MACs and from 6 SIVagm-infected AGMs, respectively. Relative transcript levels are represented by box plots in a log scale. BI indicates the baseline before infection (n = 42 corresponding to 7 time points for each of the 6 animals) and the following boxes present the gene expression after infection (n = 6 per box). The top and the bottom of the boxes represent the 75^th ^and 25^th ^percentiles, respectively, whereas the horizontal line between the box limits represent the median. Open circles indicate individual values which are not included between the 90^th ^and 10^th ^percentiles. Dark and light grey boxes indicate significant (p < 0.05) increases and decreases, respectively, relative to the baseline. Stars indicate a trend towards significant up-regulation (p < 0.08). The data on AGMs (*tnf-α*, *ifn-γ *and *il-10*) were previously published [1]. The latter are displayed here in a log scale to allow easy and direct comparisons with the data obtained for the pathogenic SIVmac infection.

We quantified the expression profiles of pro- and anti-inflammatory factors (*tnf-α*, *ifn-γ *and *il-10*) starting from 24 h after SIVmac infection. We compared them to those in non-pathogenic SIVagm infection, at the same time points using the same tools. Significant *tnf-α *up-regulations in MACs' PBMC were detected from days (d) 3 to 10 and at d28 p.i. (p ≤ 0.046). *Ifn-γ *gene up-regulations were observed from d1 to d16 p.i. (p ≤ 0.021) (Figure 1A, upper panels). In contrast, the *il-10 *gene expression was significantly down-regulated during the first 2 weeks p.i. (p ≤ 0.025) and was significantly up-regulated only at day 28 p.i. (p = 0.0003). This is in line with the previously reported profile of IL-10 concentrations in plasma from the same animals [1] and with the report of maximal increase of IL-10^+ ^cells in lymph nodes at day 28 p.i. [12]. SIV-infected AGMs exhibited no *tnf-α *increase, a later and more transient *ifn-γ *up-regulation (d10-16 p.i.), and an earlier upregulation of *il-10 *expression (d6-16 p.i.) as previously reported [1] (Figure 1A, lower panels). These data confirm a distinct early pro- and anti-inflammatory balance between these pathogenic and non-pathogenic SIV infections.

In order to search for further early differences, we quantified the transcript levels of *t-bet *and *gata-3*, which encode for essential transcription factors for the commitment towards Th1 and Th2 responses, respectively [13,14]. PBMC of SIVmac-infected MACs displayed significant increases of *t-bet *at d3-10 and 28 p.i. (p ≤ 0.017), whereas SIVagm-infected AGMs displayed either no change or even decreases in *t-bet *(d1, d6 p.i.), (p ≤ 0.044) (Figure 1B). Regarding *gata-3 *expression, we observed significant increases during both SIVmac and SIVagm infections (p ≤ 0.027). The difference between these both infections consisted in the lack of induction of Th1-associated transcription factor in AGMs.

**Figure 2:**
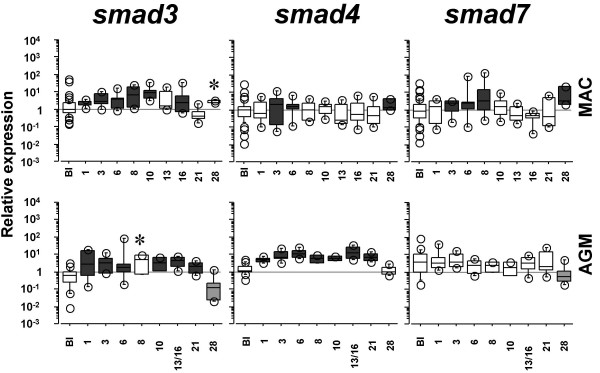
**Dynamics of *smad3*, *smad4 *and *smad7 *expressions in PBMC during pathogenic and non-pathogenic SIV infections**. See legend in Figure 1.

The expression of T-bet is known to be suppressed by TGF-β1 [15]. The latter plays indeed a major role in the negative regulation of inflammation. To assess whether AGMs and MACs might differ in their capacity to respond to TGF-β1, we analysed the expression of Smads which are the major established intracellular effectors of the TGF-β1 signaling pathway [16]. They comprise three subgroups: receptor-regulated Smads, common Smads and inhibitory Smads. We measured the gene expression of one Smad from each group, respectively, *smad3*, *smad4 *and *smad7*. Smad3 and 4 are known to activate the TGF-β1 signaling cascade whereas Smad7 inhibits the TGF-β1 signaling. We detected an up-regulation of *smad3 *starting from d1 p.i. until the 3rd week p.i. in both models (p ≤ 0.008) (Figure 2). In contrast, *smad4 *up-regulation was more transient in MACs (p ≤ 0.0023) than in AGMs (p ≤ 0.028), where it persisted for 3 weeks (Figure 2). *Smad7 *was up-regulated during primary SIVmac infection at d1-8 and 28 p.i. (p ≤ 0.026) (Figure 2). In contrast, AGMs did not display any increase and even exhibited a significant decrease of *smad7 *expression at d28 p.i. (p = 0.004).

## Discussion

These data confirm that the early cytokine balance is different between pathogenic SIVmac251 and non-pathogenic SIVagm.sab infections: more towards inflammatory responses in the former and more towards anti-inflammatory responses in the latter. Our data on Smads suggest that after SIV infection, AGMs are able to respond to TGF-β1 whereas MACs cannot, due to the up-regulation of *smad7 *gene expression and to the lack of sustained up-regulation of *smad4 *compared to the AGMs. This might explain why AGMs are more able to rapidly control the virus-driven inflammation/activation than MACs.

Mice suffering from inflammatory bowel disease (IBD) caused by an infectious agent, *Toxoplasma gondii*, display up-regulations of *smad7 *and *t-bet *gene expressions in CD4^+ ^T cells from the *lamina propria *[17]. Overexpression of Smad7 and unresponsiveness to TGF-β1 also characterized *lamina propria *mononuclear cells in gut from patients suffering from Crohn's disease [18]. Here our study reports such increases of *t-bet *and *smad7 *during acute SIVmac infection in MACs but interestingly not during acute SIVagm infection in AGMs. This may be relevant for HIV infection, where the intestinal mucosal system is an early major viral target [19], and where expression of inflammatory factors correlates with disease progression [20].

The increase of *smad7 *in SIVmac-infected MACs might take place in infected cells and/or be due to indirect mechanisms, such as the strong induction of *ifn-γ *which is known to act as a positive regulator of *smad7 *gene expression [21]. *Ifn-γ *is more increased in early SIVmac infection than in SIVagm infection. SIVmac itself might dysregulate the TGF-β1 signaling cascade by interacting directly or indirectly with Smad molecules. Indeed, HCV and HTLV-1, which also mediate chronic viral infections, were reported to do so [22-25]. For instance, the HTLV-1 Tax protein is able to abrogate interactions of Smad3 and Smad4 with cellular transcription factors [22,24,25].

TGF-β1 can negatively regulate activation through Treg induction [26-28], among other mechanisms. Recent studies have highlighted the important role of TGF-β1 responsiveness not only for the induction and stabilization of regulatory activity of CD4^+^CD25^+ ^Treg but also for the capacity of other cells to respond to CD4^+^CD25^+ ^Treg activity [14,26-29]. In a model of IBD in mice, conventional activated T cells which do not respond to TGF-β are not controlled by functional Foxp3^+ ^Treg and a dramatic accumulation of activated IFN-γ^+^CD4^+ ^T cells is observed in the gut [29]. HTLV-1^+ ^patients suffering from tropical spastic paraparesis have decreased frequencies of Foxp3^+^CD4^+^CD25^+ ^Treg as well as impaired Treg functions [30,31]. It is so far unclear if this impairment of Treg function is due to the ability of Tax to inhibit the TGF-β1 signaling cascade.

The role of Treg during HIV/SIV infections is still controversial. Some studies propose a negative effect of Treg as they suppress effector T cell responses [12,32-34]. Others provide evidence associating Treg with a favorable outcome of the infection and suggest that they are beneficial by preventing harmful generalized T cell activation [1,35-38]. In HIV/SIVmac infections, high VL in lymphoid tissues is associated with chronic and generalized T cell activation. HIV-1^+ ^patients exhibit accumulation of Foxp3^+ ^Treg in tonsils in correlation with their viral load [33]. SIVmac-infected MACs display in their lymph nodes (LN) an increase of TGF-β1^+^Foxp3^+^CD25^+^CD4^+ ^cell numbers (d7-d28 p.i.) concomitantly with an elevation of VL [12]. These putative CD4^+ ^Treg are however not capable of limiting the massive T cell hyperactivation in LN [12]. It was suggested that HIV-specific CD25^+ ^Treg cell function is compromised relatively early in HIV disease [37]. The Treg functions and/or the capacity of conventional activated T cells to respond to TGF-β1 (i) may vary between progressors and long-term non-progressors after HIV/SIVmac infections and (ii) could contribute to the balance between HIV-specific effector responses and harmful generalized T cell activation. In the future, it will be important to study the capacity of conventional activated T cells and of Foxp3^+^Treg from HIV-infected individuals to respond to TGF-β1. The capacity to respond to TGF-β1 might be an important determinant, among others virus-host determinants, i.e. the level of Nef-mediated downregulation of CD3 [39] or the levels of Siglec expression [40], for the levels of T cell activation and thus for the outcome of HIV/SIV infections.

To conclude, in response to SIV infection, our study reveals increases of *smad7 *expression in MACs as compared to AGMs. The latter retain longer lasting *smad4 *expression, in conjunction with earlier TGF-β1 and IL-10 induction. Our study suggests that differences in the capacity to control harmful inflammation in non-pathogenic and pathogenic infections are associated with differences in the early activation or inhibition of the TGF-β1 signaling pathway.

## Competing interests

The author(s) declare that they have no competing interests.

## Authors' contributions

MJYP performed total RNA extractions from African Green Monkeys' PBMC, reverse transcription of total RNA from African Green Monkeys and Rhesus Macaques, real time PCR assays, statistical analysis, participated in discussions of experimental design and writing of the manuscript. JFD and PRS equally contributed to total RNA extraction from Rhesus Macaques' PBMCs and participated in discussions of experimental design. IP participated in discussions of the experimental design. OMD performed SIVagm infections, follow-up of African Green Monkeys and contributed to experimental design. AH contributed to experimental design and critical reading of the manuscript. CB was responsible for the follow-up of macaques and contributed to experimental design. FBS contributed to experimental design. MCMT supervised experimental design and writing of the manuscript.
